# The Nutritional Behaviour of Pregnant Women in Poland

**DOI:** 10.3390/ijerph16224357

**Published:** 2019-11-08

**Authors:** Katarzyna Ługowska, Wojciech Kolanowski

**Affiliations:** Faculty of Medical and Health Sciences, Siedlce University of Natural Sciences and Humanities, Siedlce PL-08-110, Poland; katarzyna.lugowska.zdoz@uph.edu.pl

**Keywords:** diet, maternal nutrition, nutritional behaviour, pregnancy

## Abstract

A woman’s diet during pregnancy can significantly affect her health, as well as her child’s future development and well-being. Unfortunately, many pregnant women do not follow the recommended nutritional guidelines. The reason could be that they have insufficient knowledge about nutritional best practice. Accordingly, the purpose of this study is to investigate the nutritional behaviour of pregnant women in Poland. The research was conducted using a questionnaire to survey a sample of *N* = 815 pregnant women in first pregnancy. Among the findings were that the subjects ate an excessive amount of sweets, and white bread, and consumed insufficient quantities of fish, milk and fermented milk drinks. Subjects chose white bread more often than wholemeal bread, and fruit rather than vegetables. The study showed that the nutritional behaviour of pregnant women was characterised by many bad practices.

## 1. Introduction

The diet of a woman during pregnancy is one of the most important factors affecting the health of the mother and child. Epidemiological studies have shown that the prenatal period of development largely determines the occurrence of lifestyle and civilization diseases in later life [1,2,3,4]. Proper preparation of a woman for pregnancy has a significant impact on embryo genesis. The occurrence of perinatal complications and malformations is often associated with metabolic abnormalities and nutrient deficiencies in the mother. These can also be caused by excessive exposure to some chemicals, including those present in food [5]. According to the theory of metabolic programming, environmental factors and lifestyle, including diet, during the pregnancy of the mother, among other effects, has an influence on the health and disease risk in the later life of the offspring [6]. Obesity or malnutrition in a future mother is considered to be one of the most important factors determining intrauterine fetal development [7,8]. Elimination diets, which have recently been fashionable among young mothers, may cause nutritional deficiencies, which in turn can lead to complications with the pregnancy. Currently, more and more people are paying attention to healthy eating. This phenomenon is linked to the increasing awareness of consumers about environmental and health issues [9,10,11].

Pregnant women should consume a variety of foods to maintain a healthy body weight [12], and to meet the demand for energy and nutrients, which are associated with the formation of fetal and maternal tissues [13,14,15,16,17]. Metabolic shift in pregnancy is associated with the accumulation of energy reserves for the period of puerperium and lactation, and also provides the right amount of oxygen and nutrients for the child’s development [18].

Nutrients in a women’s diet are important for a child’s development during gestation, particularly: folic acid, calcium, iodine, iron, zinc and long-chain polyunsaturated omega-3 fatty acids [19,20]. Deficiencies can often occur during this period. Dietary supplementation with folic acid, both before pregnancy, and in the first weeks of gestation, is recommended. Adequate folic acid supply protects against megaloblastic anaemia and prevents neural tube defects in children [21]. Insufficient iron supply increases the risk of premature delivery and low birth weight. Lack of iodine in the diet increases the risk of mental retardation and thyroid disease. Insufficient calcium intake may cause gestational hypertension, reduced bone density in the new-born, and increase the risk of premature delivery. During pregnancy, not only deficiencies, but also excessive amounts of certain substances, especially vitamins A and D, can be dangerous. The conditions for the proper course of pregnancy include the mother’s knowledge, not only of the principles of healthy eating, but also how improper nutrition can negatively influence the development of the child and the course of the pregnancy [22,23,24].

A mother’s behaviour, before and during pregnancy, is of great importance, both for her own health, and also that of her child [24,25]. Inadequate knowledge of nutritional principles can result in many dietary errors, which may result in an excess or deficiency of energy and specific nutrients that are important for the successful course of a pregnancy and a child’s healthy development [26,27]. Good nutrition, combined with appropriate physical activity and avoidance of bad habits, increases the likelihood that the course of the pregnancy will follow an optimal course [28,29].

The aim of this study was to assess the nutritional behaviour of pregnant women in Poland. The working hypothesis assumed that despite universal access to information on the principles of a healthy diet and the impact of diet on pregnancy, and the health of a mother and child, most pregnant women do not follow the relevant recommendations.

## 2. Materials and Methods

The research was carried out among *N* = 815 women all in first pregnancy, from January 2017 to March 2019 using a specially designed questionnaire. The questionnaire was sent to a hospital located in Siedlce (Poland), including opinions in this case, remarks and suggestions used to apply the refinement of the survey. The survey was updated on the basis of feedback received, such as: gestational age, mother’s state of health, order of pregnancy, occurring pathological conditions. The questionnaire was validated during a pilot study conducted in 2016 among a group of pregnant women (50 women).

### 2.1. Procedure

Consent to participate in the study was accepted as an inseparable element for respondents who completed the questionnaire voluntarily. The study design obtained written approval of the Ethics Committee in Scientific Research of the Siedlce University. The questionnaire was distributed by email and links to websites from which the study participants were invited. Women were also invited to participate in the study through social networking sites and websites devoted to pregnant women. Participants provided secret consent by opening the link and by filling out the questionnaire. The survey contained questions related to the course of pregnancy, selected eating behaviours and attitudes regarding the frequency of food consumption, as well as socio-demographic characteristics; it did not focus on the above-mentioned elements covering the pre-pregnancy period.

### 2.2. Inclusion/Exclusion Criteria

Participants were randomly selected, provided the patient met the selection criteria: (i) first pregnancy; (ii) pregnancy of normal course and duration >14 weeks; (iii) the woman’s age > 19; (iv) place of residence—Poland; (v) no participation in antenatal classes and educational lessons; (vi) answering all questions; (vii) consent to participate in the study. In addition, the exclusion criteria were risk factors such as miscarriage, second or next pregnancy, pregnancy-induced hypertension and gestational diabetes, of which women were informed prior to the survey.

### 2.3. Questionnaire

The questionnaire contained 28 multi-choice questions with one answer to choose from for each question. Participants were not limited in time. Each of the participants was informed about the purpose of the study, and assured of the anonymity of their data, before completing the questionnaire. In addition, an information sheet was provided at the beginning of the questionnaire containing information about the study and contact details for additional information.

The first part of the questionnaire included questions about pregnancy and its course (such as: mother’s age, gestational age, health condition, order of pregnancy, occurring pathologies), as well as socio-demographic information (such as: education, place of residence, financial situation). The study assumes three levels of financial standing (above average, average, below average) and two levels of education: lower (such as: primary or secondary education hereinafter referred to as basic education) and higher (such as: student and higher) and places of residence: town and village.

The second part of the questionnaire contained questions about the frequency of eating basic food types (such as meat, poultry, fish, bread, cereal, pasta, vegetables, fruit, nuts, sweets, fast-food), the number of meals consumed during the day, and the breaks between them. Participants were also asked questions about their own nutritional knowledge, as well as about any physical activity being undertaken.

### 2.4. Statistics

The data obtained was entered into a Microsoft Excel 2010 (Microsort, Redmond WA, USA) spreadsheet and summarised. Then, statistical analysis was performed using StatSoft Statistica v. 12 (StatSoft, Tulsa OK, USA)), using the non-parametric test χ^2^. A significance level of α = 0.05 was assumed for all subjects. It was assumed that for values in which *p* < 0.05 or equal to 0.05 there are statistically significant differences, while for *p* > 0.05 the variables can be considered statistically insignificant.

## 3. Results

The participants of the study were adult women with normal pregnancy and a gestational age >14 weeks of pregnancy. All women lived in Poland (Table 1).

The majority of the subjects were city dwellers (64%), the remainder lived in rural areas (36%). Among the participants, 44% had completed higher education, and 56% had only primary or secondary education, hereinafter referred to as basic education. Questioned about their financial situation, 77% of the subjects described it as average, 16% as above average, and 7% classified it as below average (Table 1)

**Figure 1:**
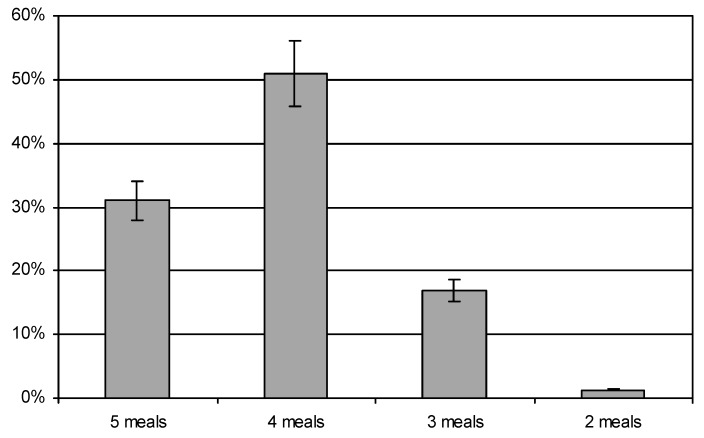
Number of meals eaten during the day eaten by participants.

There were no statistically significant differences in the results relating to the place of residence (larger and smaller towns). However, some variation was shown in relation to the level of education, which is discussed later in this paper. Most of the subjects reported eating four (51%) or five meals a day (31%) (Figure 1). However, some women, especially those with a basic education, ate only three, or less meals a day—17% or less 1%, respectively (χ^2^ = 26.56250, df = 4, *p* < 0.05).

Regular eating at a fixed time was indicated by 19% of the subjects with higher education, and 8% of those with basic education. The differences between these two groups were statistically significant (χ^2^ = 65.59705, df = 2, *p* < 0.05).

**Figure 2:**
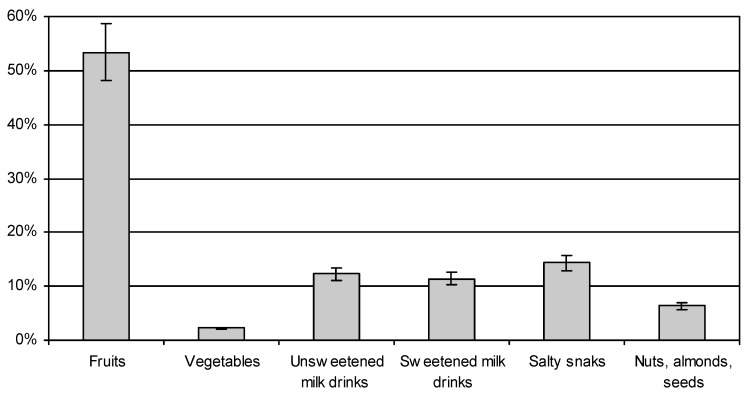
Type of food eaten between main meals by participants.

Women with higher education reported eating between meals most often several times a week (32%), or once a day (31%); those with basic education reported snacking several times a day (36%), and once a day (32%). Women with basic education ate more frequently between meals (*p* < 0.05). The highest number of women, both those with higher education, and those with basic education, reported that between meals most often they ate fruit (53.43% on average), followed by salty snacks (14.31%) and unsweetened milk drinks and desserts (12.27%) (Figure 2). A much larger number of pregnant women with basic education reached for salty snacks (18.88%), and sweet dairy desserts (12.5%) compared to women with higher education, where the consumption of these products averaged only 10%. Vegetables and nuts were consumed relatively less frequently between main meals. A small number of subjects ate vegetables and nuts between main meals (2.60% vegetables and 8.12% nuts of those with higher education; and 4.59% nuts and 1.79% vegetables of those with basic education) (χ^2^ = 48.09833, df = 5, *p* < 0.05).

**Figure 3:**
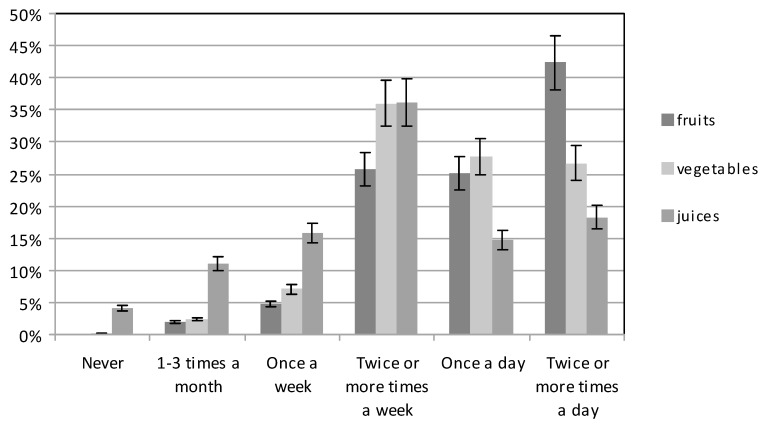
The consumption of fruit, vegetables, and juices by participants.

Among the beverages drunk by pregnant women, both those with higher and those with basic education, still water was the most frequent choice (78.57% and 72.45%, respectively), while sparkling water was preferred by 12.52% of the women in both groups. Women with basic education more often drank flavoured water (12.24%) than women with higher education (3.25%) (χ^2^ = 36.90951, df = 3, *p* < 0.05). Fruit and vegetable juices were consumed relatively infrequently (Figure 3). Women with higher education usually chose them several times a week (34.09%) and once a day (17.86%), while they were chosen by those with basic education several times a week (38.27%) and several times a day (18.88%). Only 4.89% of the subjects with higher education and 3.32% of those with basic education did not drink fruit and vegetable juices at all (χ^2^ = 30.18917, df = 5, *p* < 0.05).

Vegetables and fruit were eaten at different frequencies (Figure 3). The largest number of women with higher education consumed vegetables several times a week (33.77%), and several times a day (33.44%); those with basic education mostly ate vegetables several times a week (38.27%), or once a day (28.06%) (χ^2^ = 53.82904, df = 5, *p* < 0.05). Fruit was eaten quite frequently by both groups. Pregnant women with higher education and those with basic education reported eating fruit most often several times a day (47.73% or 36.99%, respectively). Eating fruit at least once a day was reported by 28.57% of the women with higher education, and 21.68% of those with basic education, while the figures, for those who only ate fruit several times a week, were 18.83% and 32.65%, respectively (χ^2^ = 49,199,250, df = 5, *p* < 0.05).

**Figure 4:**
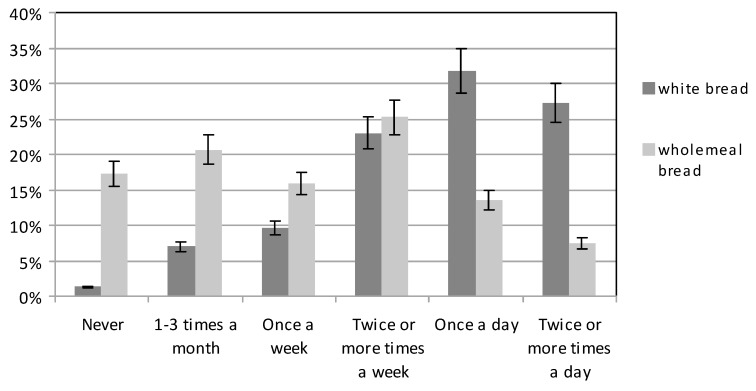
The consumption of white and wholemeal bread and other cereal products.

**Figure 5:**
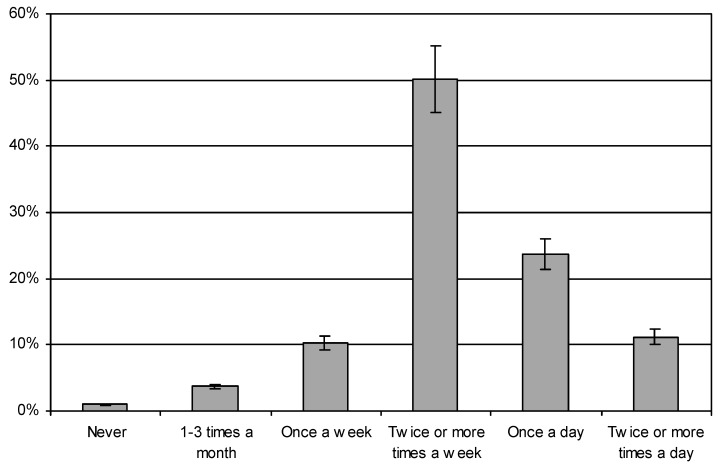
The consumption of milk products by participants.

Participants were more likely to consume white bread rather than wholegrain bread; those with basic education slightly more often (*p* < 0.05) (Figure 4). In contrast, the largest number of women with higher education, 32.79%, consumed wholegrain bread several times a week, while only 17.60% of those with basic education ate wholegrain bread several times a week. On average, 21% of the women reported eating wholegrain products at least once a day; 25% of those with higher education and 17% of those with basic education. In contrast, on average 15.93% of the subjects consumed wholegrain bread only once a week; 12.99% with higher education and 18.88% with basic education (*p* < 0.05) (Figure 5). It is notable that on average over 17% of the women did not eat wholegrain products at all: 10.39% of women with higher education and 24.23% with basic education.

Both groups of women most frequently used butter to spread on their bread; 58.44% of those with higher education and 50.26% of those with basic education. Fat mixes and margarine were used much less frequently. Those with lower education more often spread butter substitutes on bread (23.47% mixes and 17.35%, margarine) than women with higher education (11.69% and 14.61%, respectively). 9.74% of the women with higher education and 4.34% of those with basic education did not spread any fat at all on their bread (χ^2^ = 53.60788, df = 4, *p* < 0.05).

Participants reported that quite frequently they ate white rice, plain pasta, and fine-milled cereals. Both groups of women, those with higher education, and those with basic education, consumed them several times a week (39% of each groups) or once a week (33.12% and 31.89%, respectively). A significant percentage of the participants (23.35%) ate such products only 1-3 times a month (χ^2^ = 18.66961, df = 5, *p* < 0.05).

Subjects also reported relatively low consumption of wholemeal cereals, brown rice, oatmeal and wholegrain pasta. However, significantly women with higher education consumed such dishes more often than those with basic education. Both groups reported that most often they ate such products several times a week (24.68% of those with higher education, and 21.17%, of those with basic education), or once a week (20.78% and 17.86%, respectively). Subjects who did not eat these foods at all comprised 11.36% of those with higher education, and 22.45% of those with basic education (χ^2^ = 77.40367, df = 5, *p* < 0.05).

On average, half the group drank milk and fermented milk drinks several times a week (Figure 5). Significantly more women with higher education (39.94%) consumed these drinks at least once a day, than those with basic education (29.85%). Some 5% of women with higher education, and 15% of those with basic education, drank them only once a week. (χ^2^ = 47,60925, df = 5, *p* < 0.05).

**Figure 6:**
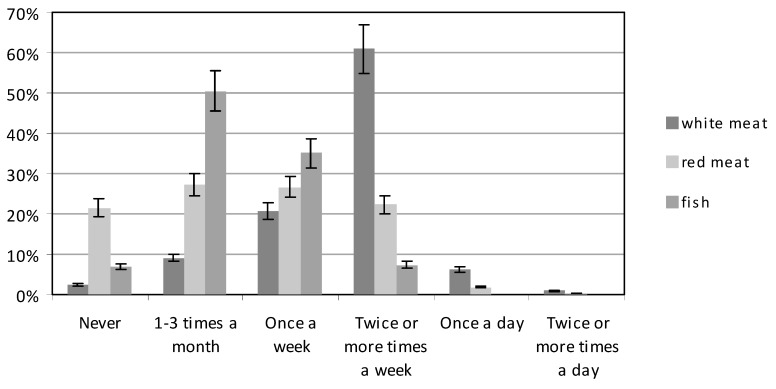
The consumption of meat and fish by participants.

The frequency of consumption of meat and meat products by each group was similar (Figure 6). Most women ate meat and meat products, either 1-3 times a month (approx. 27% both groups), or once a week (approx. 27% both groups) (χ^2^ = 23.76171, df = 5, *p* < 0.05). Red meat was consumed several times a week by a larger number of women with higher education (24.35%), than by those with basic education (20.15%). However, 21.47% of women did not eat red meat at all. White meat (chicken, turkey and other poultry) was consumed several times a week by the largest number of women, both those with higher education (57.79%), and those with basic education (64.03%). White meat was consumed only once a week by 21.75% and 19.39% respectively of the two groups (Figure 6). Only 6.82% of the subjects with higher education, and 5.61% of those with basic education, consumed white meat once a day (χ^2^ = 23.18648, df = 5, *p* < 0.05).

The women ate fish relatively infrequently (Figure 6). Both groups most often ate fish 1-3 times a month; 48.70% of those with higher education, and 52.30% of those with basic education. A significantly higher percentage of women with higher education consumed fish once a week (40.26%) compared to women with basic education (29.85%) (*p* < 0.05). Some 3.90% of the women with higher education, and 10.20% of the women with basic education, did not eat fish at all (χ^2^ = 36.90823, df = 4, *p* < 0.05).

**Figure 7:**
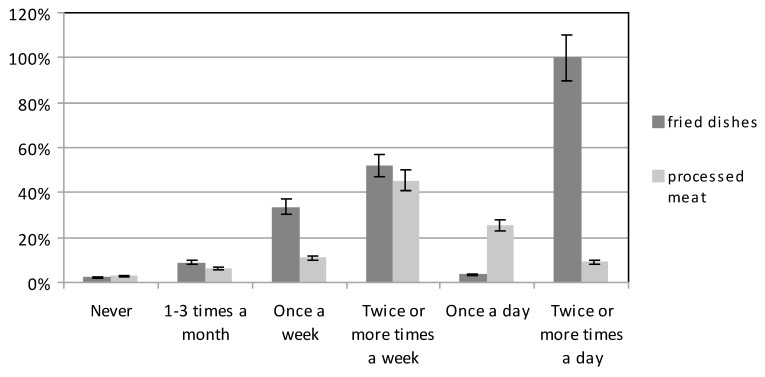
The consumption of fried dishes and processed meat products by participants.

The consumption of smoked meat products by both groups was at a similar level (Figure 7). Some 44.81% of women with higher education most frequently consumed such products several times a week, as also did 45.92% of those with basic education. Some 11.69% of women with higher education, and 10.20% of those with basic education, consumed smoked meats only once a week. Smoked meats were not consumed at all by 3.25% of women with higher education, and 2.55% of those with basic education (χ^2^ = 23.40301, df = 5, *p* < 0.05).

Meals containing fried dishes were eaten quite frequently (Figure 7). About half of the women, both those with higher education, and also those with basic education, consumed such meals several times a week (48.70% and 55.10%, of the two groups); one-third ate them once a week (33.70% and 33.16%, respectively); 3.25% and 3.83% respectively, ate them daily; and 3.57% and 0.77% respectively, did not eat fried foods at all (χ^2^ = 49.25952, df = 5, *p* < 0.05).

**Figure 8:**
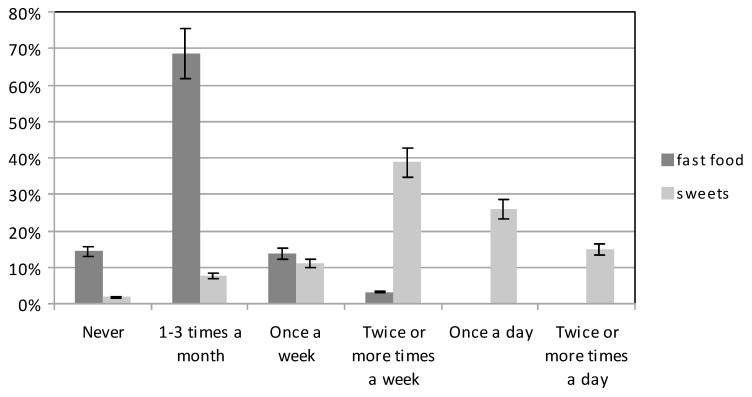
The consumption of fast food and sweets by participants.

Fast food dishes are not recommended in the diet of pregnant women, and the participants ate these only infrequently. However, sweets and confectionery products were eaten quite frequently (Figure 8). Sweets were eaten several times a week by 39.61% of the group with higher education, and 37.76% of the group with basic education. The respective group percentages for sweet consumption at a frequency of once a day were 25% and 26.79%. The group percentages for women who consumed sweets several times a day were 13.96% and 15.82%, respectively (χ^2^ = 49.76262, df = 5, *p* < 0.05).

**Figure 9:**
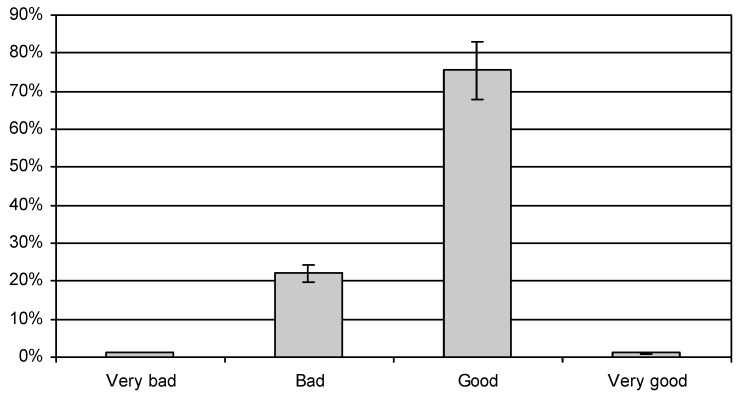
Participants own assessment of diet quality.

When assessing the quality of their own diet, 75.57% of the women described it as good (Figure 9), 22.09% of women rated it as bad, while only 1% thought that they were eating very well (χ^2^ = 31.87428, df = 3, *p* < 0.05). As regards their nutritional knowledge, 85.71% of the women with higher education, and 85.20% of those with basic education described it as average. The percentages of both groups which assessed it as above average (8.06%) or as below average (6.48%) were the same. (χ^2^ = 26.61005, df = 2, *p* < 0.05).

In addition to dietary behaviour, the level of physical activity was also investigated. The majority the subjects (59.83%) assessed their level of physical activity as moderate. While only 3.90% of the women with higher education and 2.55% of those with basic rated it as high (χ^2^ = 20.16056, df = 2, *p* < 0.05).

## 4. Discussion

Analysis of the data highlighted instances of inappropriate nutritional behaviour, both among the women with higher education and also among those educated to a basic level. Nevertheless, on average, women with higher education exhibited better nutritional behaviour than women with basic education. The results of our research are also confirmed by other authors. A similar finding was also obtained by the multidimensional data analysis of Desta et al. (2019), covering a group of 315 pregnant women. In that study, level of education was significantly related to diet choices [30]. In addition, a study by Aynaci (2019) found that women with higher education had a better knowledge of the principles of a healthy diet [31]. Similar results were obtained in the study of Most et al. (2019), which found the nutritional behaviour of a group of women who had completed their higher education to be better than of those who had only basic education [32].

Nutrition standards for the Polish population, reflect World Health Organization (WHO) and European Food Safety Authority (EFSA) recommendations [33,34]. Similar standards have been published by the Institute of Medicine [35].

The demand for energy, nutrients and fluids increases during pregnancy. These result from higher resting energy expenditure. During pregnancy, the body uses more energy for growth (foetus and placenta) and more intensive work of the lungs and heart. In Polish standards, the energy of the diet should be increased by 360 kcal/day in the second trimester of pregnancy and 475 kcal/day in the third trimester [34]. International recommendations suggest that during pregnancy the daily increase of energy intake should be 85 kcal per day in the first trimester, 285 kcal per day in the second trimester and 475 kcal per day in the third trimester [36].

The increased requirements for nutrients during pregnancy also applies to most vitamins and minerals. It is recommended to eat meals regularly and compose them using products from all groups (vegetables and fruit, cereal, dairy, meat, fish, eggs, vegetable oils, nuts and legumes) [37].

This study shows that a significant proportion of the subjects ate 4 meals a day, while 31% ate 5 or more meals a day. Nutrition standards for the Polish population recommend eating 4–5 meals a day. These values correspond to 1–2 additional snacks or an additional meal. The study of Desta et al. (2019) showed better behaviour, because nearly 77% of the women indicated that they consumed four or more meals a day, 23% consumed 1–3 meals a day, while 21% had the habit of eating between meals [30]. Pieszko et al. (2017) obtained slightly more favourable results. Those studies showed that 26% of the pregnant women ate 6 meals, and 42% ate 5 meals a day [38]. Also, in the study by Dereń et al. (2017) significant percentages of the pregnant women consumed 5–6 meals (51%) or 3–4 meals (43%) during the day. In that study, 81% of women admitted to eating between meals [13]. In our research, however, as many as 98% of women ate between meals. In the Suliga study (2015), almost half of the respondents (47%) consumed 5 or more meals, and 35% ate 4 meals a day [39].

The importance of vegetables and fruit in the diet of pregnant women is significant. They provide a large amount of antioxidants (vitamin C, carotenoids, vitamin E, flavonoids), folates, potassium and fibre. According to Polish recommendations, in the first trimester pregnant women should include vegetables and fruit in the amount of 400 g and 300 g per day, and in the second and third trimester—500 g and 400 per day, respectively. Also, WHO recommends increasing the intake of vegetables and fruit in the diet of pregnant women.

In this study, women mostly consumed: fruit, salty snacks, dairy sweet drinks and desserts between meals, and much less frequently: nuts and vegetables. However, salty snacks, dairy sweet drinks and desserts have typically been characterised as poor in nutritional value, consisting mainly fat and sugars. Similar results were obtained in the study by Dereń et al. (2017), where more than half of the women consumed fruit between meals, and almost 1/3 reached for sweets [13]. In the study of Kobiołka et al. (2015), fruit was the most frequently chosen snack eaten by pregnant women between meals [40].

According to nutritional recommendations, fats should provide 20–35% of daily energy requirement. Fat consumption in the second trimester of pregnancy should be increased by an additional 8–14 g per day, while in the third trimester of pregnancy by 11–18 g per day in relation to consumption before pregnancy. Saturated fatty acids should be consumed in the smallest possible amounts [34]. Pregnant women should consume predominantly vegetable fats containing essential unsaturated fatty acids. The source of polyunsaturated fatty acids from the omega-6 family are oils: sunflower, corn, soy, evening primrose, while the source of omega-3 fatty acids are fatty fish such as herring, mackerel, salmon and rapeseed oil, walnuts, linseed [41]. Pregnant women should more often use fats that are a source of omega-3 acids. Hard margarine, fast food and processed products such as chips, powdered soups, cakes, cookies and sweets should not be consumed by pregnant women at all. They are the main sources of trans fatty acids, which can disrupt the fetal development and increase blood cholesterol [34].

Pregnancy is a period when the body’s demand for long-chain polyunsaturated omega-3 fatty acids increases, for which salt-water fish are a good source. Edible fats should include easily digestible and the least processed fats. Among the spreadable fats, butter is a good example. The results of the study showed that a significant proportion of respondents 1/2 spread butter on bread and rolls, almost 1/5 used a mix of butter with margarine, and a little bit less—margarine. In the study of Hyżyk and Sokalska (2011), 66% of pregnant women used butter for spreading on bread, and 20% used margarine [42]. In the study of Kobus-Ciskowska et al. (2016) as many as 95% of the pregnant women indicated butter as the fat they most commonly used [43].

According to the European Food Safety Authority Scientific Committee [33], carbohydrates are basic components in the maternal diet and should represent between 45% and 60% of the calories in a healthy diet in the adult population as well as during pregnancy. However, no more than 10% of energy should come from sugars. The amount necessary to maintain the proper functioning of the nervous system and homeostasis of the body and the proper development of the foetus (RDA) is 175 g per day [34]. In general, starchy carbohydrates (pasta, potatoes, bread, cereals and rice) and fibre containing whole grain cereals and vegetables are the core of a healthy diet. According to WHO/FAO recommendations, 25 g of fibre a day allows for proper functioning of the body [44].

Pregnant women should include wholegrain cereal products in their daily diet because of their much higher content of minerals, vitamins and dietary fibre compared to white bread. Although products from this group should be the basic source of energy and should be included in every main meal, 2/3 of Polish women in pregnancy consume less than 4 portions of these products during the day [39]. Study also shows a low proportion of whole grains in the diet of pregnant women. Our study showed that pregnant women were much more likely to choose white bread than wholegrain bread, which is a negative phenomenon. Only 1/5 of the subjects used wholegrain bread once or several times a day. Przybyłowicz et al. (2012) found a higher share of wholegrain bread consumption [45]. The study of Hyżyk and Sokalska (2011) or Dereń et al. (2017) showed results similar to the data obtained in this study [13,42]. The recommended consumption of wholegrain products (cereal, bread, whole meal pasta), in at least 3 meals a day, was indicated by only 15% of the respondents [13].

Dairy products are rich in calcium and riboflavin. Recommended calcium intake varies between countries. The FAO/WHO recommend a dietary intake of 1200 mg/day of calcium for pregnant women [46]. In Poland, the recommended calcium intake (RDA) is 1000–1300 mg per day for pregnant women. To cover high demand, a pregnant woman should drink 3–4 glasses of milk or fermented milk drinks (yogurt, kefir) during the day. They should choose skim milk, because the fat contained in fat milk and its products (fat yogurt, fat cheese) provides a lot of calories and promotes the development of atherosclerosis [34].

Pregnant women are recommended milk and milk products, especially fermented milk drinks, in the diet of pregnant women. Many medical associations recommend frequently milk and milk products, especially fermented milk drinks, in the diet of pregnant women. They contribute wholesome protein, vitamin B and calcium to the diet, and in the case of fermented milk products, probiotic bacteria. Our study showed a low share of milk and dairy products in the diet of pregnant women. In our study, 2/3 of the subjects reported drinking milk and fermented milk drinks at least once a week, and 1/3—at least once a day. In the Abd-Elmohda study (2019), slightly better results were obtained, almost half of the pregnant women (49%) drank the above-mentioned products once a day, and 41% at least twice a day [47]. Similarly, in the study by Dereń et al. (2017), 43% of pregnant women consumed dairy products once a day, and 48% more often [13]. Similar results were also obtained in the studies of Kobus-Ciskowska et al. (2016) and Suliga (2015) [39,43].

During pregnancy, the need for omega-3 essential fatty acids increases significantly. Polish recommendations for omega-3 docosahexaenoic acid (DHA) + eicosapentaenoic acid (EPA) consumption during pregnancy are: 250 mg EPA + 100–200 mg DHA per day, preferably in the form of oily fish servings [34]. Poland is a country of very low consumption of fish and seafood—ca. 5–6 kg (edible parts) per year [48]. This was also shown in ours study. Poles mainly consume small amounts of sea fish, such as pollock, herring, mackerel, cod, and freshwater fish from panga [49].

Fish are a source of wholesome protein, many minerals, vitamins and long-chain polyunsaturated omega-3 fatty acids, including DHA which is particularly important for brain and eye development. The recommended intake is at least 2–3 servings per week. Our study found fish consumption was too low. Slightly more than half of the subjects ate fish only 1–3 times a month, 1/3—once a week, while the remaining subjects ate them less often, or not at all. Godal’s study (2012) also showed low fish consumption, only 1/3 of the pregnant women consumed them several times a week [50]. Equally low fish consumption was presented in the Suliga study (2015) [39]. Slightly better results were obtained in the study by Dereń et al. (2017) and Kobus-Ciskowska et al. (2016), in which two-thirds of the pregnant women ate fish once a week [13,43]. In the Abd-Elmohdy study (2019), half of the subjects consumed fish occasionally, 23% ate them 2–3 times a week [47].

International dietary guidelines recommend increased protein intake during pregnancy, especially in the second and third trimesters. Protein is needed for maternal and fetal tissue and placenta. Therefore, the daily intake should be increased by 1 g per day in the first trimester, 8 g per day in the second trimester, and 26 g per day in the third trimester of pregnancy [51]. According to Polish nutrition standards, pregnant women should consume 1.2 g of protein per each kg of body weight, or 54 to 90 g per day [34]. According to EFSA, the intake of protein during pregnancy should be 52–80 g per day. The quality of the protein consumed is also important [52]. More than half (ca. 60%) of the total amount of daily protein intake, should be of animal origin, of which primary source is milk and its products as well as meat from animals, poultry and fish. The remaining 40% should come from vegetable protein—e.g., legumes (beans, peas, broad beans or soybeans). Meat is an important source of wholesome protein, vitamin B6 and PP, zinc, as well as well-absorbed iron. During the week, meat should be replaced at least 2–3 times with fish [34].

The main source of animal protein can be lean meat and its products, skimmed milk and its products, fish, poultry and eggs. A pregnant woman should limit the consumption of pork because of the high content of saturated fat. The pregnant women in our study chose white meat (poultry) much more frequently than red meat, 2/3 of women ate white meat several times a week, and 1/5 once a week. Similar results were obtained by Ab-Elmohdy (2019) and Dereń (2017) [13,47]. Similar results were obtained in the Abd-Elmohda study (2019), where 37% of pregnant women ate white meat 3–4 times a week, 31% ate it 2–3 times a week, and 27% at least once a day [47]. In the case of red meat, our study found that subjects most frequently ate it either 1–3 times a month (27.34%), or once a week (26.65%). Only 22.25% of the women ate red meat several times a week. In the Abd-Elmohdy (2019) study, 43% of women ate red meat 3–4 times a week, 12% ate it 2–3 times a week, while 33% ate it only occasionally [47].

World Health Organization (WHO) recommends that adults consume no more than 10% of energy from free sugars [53]. Pregnant women should limit the amount of sugar, sweets and sweetened products consumed in their diet. Our subjects consumed excessive amounts of such products. As many as 40.78% of the subjects ate sweets at least once a day, and 49,8% at least once a week. Just 7.70% of the women ate sweet products only occasionally (1–3 times a month). The study by Kobus-Cisowska et al. (2016) also found a high proportion of sweet products in the diet of pregnant women. As many as 66% of the women consumed sugar and sweets at least once a day, 29% once a week, only 5% 1–2 times a month [43]. The Suliga study (2015) also showed a high consumption of sweets [39].

Our research shows that a substantial majority of the subjects did not follow the dietary recommendations for pregnant women. However, when asked to assess their own diet, 75.57% of the women considered that they were eating properly during pregnancy, 1.28% assessed their diet as very good, and 22.09% rated their dietary habits as bad.

Diet and physical activity before pregnancy and during pregnancy affects the short and long-term health of mother and child. Regular physical activity contributes to the maintenance of normal weight of the pregnant woman [54]. Nevertheless, pregnant women show usually low levels of physical activity, which was also shown in our study. Regular physical activity is known to be beneficial to both physical and mental health. The WHO recommends that adults aged 18-64 do at least 150 min of moderate intensity physical activity per week [55]. Pregnant women may exhibit sedentary behaviour and be physically inactive because of the need to adapt to significant physiological and psychological changes during pregnancy. Regular, moderate exercise is recommended unless there are medical contraindications [56]. In addition, despite the health benefits of regular physical activity during pregnancy, many women do not follow the recommended guidelines. Many studies indicate a decrease in the level of physical activity of pregnant women, this phenomenon was also observed in our study [57,58,59]. In our study, the vast majority of women (59.83%) described their physical activity as low, 36.94% as moderate, and 3.22% as high.

The study showed that the level of education had an influence on the quality of diet. It identified also which nutritional behaviours were in line with dietary recommendations and which were against the recommendations. Differences in nutritional behaviour between pregnant women with higher and basic education have been observed by many researchers. The study by Desta et al. (2019) shows that higher levels of women’s education were significantly associated with appropriate nutritional diversity in comparison with the diets of women who had only completed their basic education [30]. A possible explanation may be that those mothers who have completed university education can better understand nutritional recommendations and other educational messages delivered by various media.

## 5. Conclusions

Many bad practices were discovered in the nutritional behaviour of pregnant women, including insufficient consumption of fish, vegetables and milk and fermented milk drinks and excessive consumption of sweets and fruit. Women with higher education had a slightly higher frequency of good nutritional practice. White bread was chosen in preference to wholegrain bread. Despite this, a significant proportion of pregnant women rated their diet as good. In this era of universal access to information and developed prenatal care for women, there is still a need for education about proper nutrition and the impact of diet on the health of women and their children.

## Figures and Tables

**Table 1:** Characteristics of the group of pregnant women participating in the study.

*N*	*Age (Years)*	*Gestational Age (Weeks)*	*Place of Residence*		*Education*		*Physical Activity Level*		
	*>18*	*>14*	*City*	*Village*	*Basic*	Higher	Large	Moderate	Low
***815***	***100%***	***100%***	***64%***	***36%***	***44%***	56%	3.22%	36.94%	59.83%

## References

[1] Renault K.M., Carlsen E.M., Nørgaard K., Nilas L., Pryds O., Secher N.J., Olsen S.F., Halldorsson T.I. (2015). Intake of sweets, snacks and soft drinks predicts weight gain in obese pregnant women: Detailed analysis of the results of a randomised controlled trial. PLoS ONE.

[2] Symington E.A., Baumgartner J., Malan J., Zandberg L., Ricci C., Smuts C.M. (2018). Nutrition during pregnancy and early development (NuPED) in urban South Africa: A study protocol for a prospective cohort. BMC Pregnancy Childbirth.

[3] Malek L., Umberger W., Makrides M., Zhou J.S. (2016). Adherence to the Australian dietary guidelines during pregnancy: Evidence from a national study. Public Health Nutr..

[4] Ho A., Flynn A.C., Pasupathy D. (2016). Nutrition in pregnancy. Obstet. Gynaecol. Reprod. Med..

[5] Tanha F.D., Mohseni M., Ghajarzadeh M., Shariat M. (2013). The effects of healthy diet in pregnancy. J. Fam. Reprod. Health..

[6] Cetin I., Bühling K., Demir C., Kortam A., Prescott S.L., Yamashiro Y., Yarmolinskay M., Koletzko B. (2019). Impact of Micronutrient Status during Pregnancy on Early Nutrition Programming. Annu. Nutr. Metab..

[7] Anleu E., Reyes M., Araya B.M., Flores M., Uauy R., Garmendia M.L. (2019). Effectiveness of an Intervention of Dietary Counseling for Overweight and Obese Pregnant Women in the Consumption of Sugars and Energy. Nutrients.

[8] Englund-Ögge L., Brantsæter A.L., Sengpiel V., Haugen M., Birgisdottir B.E., Myhre R., Meltzer H.M., Jacobson B. (2014). Maternal dietary patterns and preterm delivery: Results from large prospective cohort study. BMJ.

[9] Lesser M.N.R., Mauldin K., Sawrey-Kubicek L., Gildengorin V., King J.C. (2019). The Type of Dietary Fat in an Isocaloric Breakfast Meal Does Not Modify Postprandial Metabolism in Overweight/Obese Pregnant Women. Nutrients.

[10] Zelalem A., Endeshaw M., Ayenew M., Shiferaw S., Yirgu R. (2017). Effect of Nutrition Education on Pregnancy Specific Nutrition Knowledge and Healthy Dietary Practice among Pregnant Women in Addis Ababa. Clin. Mother Child Health..

[11] Ickovics J., Lewis J., Kershaw T., Magriples U. (2017). Group prenatal care compared with traditional prenatal care—A systematic review and meta-analysis. Obst. Gynecol..

[12] Stubert J., Reister F., Hartmann S., Janni J. (2018). The Risks Associated with Obesity in Pregnancy. Dtsch. Arztebl. Int..

[13] Dereń K., Gaweł M., Łuszczki E., Jarmakiewicz S., Sokal A., Polak E., Wyszyńska J. (2017). Nutritional behavior of pregnant women from the Podkarpacie province. Eur. J. Clin. Exp. Med..

[14] Blondin J.H., LoGiudice J.A. (2018). Pregnant women’s knowledge and awareness of nutrition. Appl. Nurs. Res..

[15] Bookari K., Yeatman H., Williamson M. (2016). Exploring Australian women’s level of nutrition knowledge during pregnancy: A cross-sectional study. Int. J. Womens Health.

[16] Soltani H., Duxbury A., Rundle R., Marvin-Dowle K. (2017). Dietary habits and supplementation practices of young women during pregnancy: An online cross-sectional survey of young mothers and health care professionals. BMC Nutr..

[17] Whitaker K.M., Wilcox S., Liu J., Blair S.N., Pate R.R. (2016). Pregnant women’s perceptions of weight gain, physical activity, and nutrition using Theory of Planned Behavior constructs. J. Behav. Med..

[18] Koletzko B., Godfrey K.M., Poston L., Szajewska H., van Goudoever J.B., de Waard M., Brands B., Grivell R.M., Deussen A.R., Dodd J.M. (2019). Nutrition During Pregnancy, Lactation and Early Childhood and its Implications for Maternal and Long-Term Child Health: The Early Nutrition Project Recommendations. Ann. Nutr. Metab..

[19] Stråvik M., Jonsson K., Hartvigsson O., Sandin A., Wold A.E., Sandberg A.S., Barman M. (2019). Food and Nutrient Intake during Pregnancy in Relation to Maternal Characteristics: Results from the NICE Birth Cohort in Northern Sweden. Nutrients.

[20] Mousa A., Naqash A., Lim S. (2019). Macronutrient and Micronutrient Intake during Pregnancy: An Overview of Recent Evidence. Nutrients.

[21] Mroczek A., Bałabuszek K., Pawlicka M., Semczuk-Sikora A. (2018). Knowledge about folic acid supplementation before and during pregnancy among female medical fields students. J. Educ. Health Sport.

[22] Marangoni F., Cetin I., Verduci E., Canzone G., Giovannini C., Scollo P., Corsello G., Poli A. (2016). Maternal Diet and Nutrient Requirements in Pregnancy and Breastfeeding. An Italian Consensus Document. Nutrients.

[23] Morrison J.L., Regnault T.R.H. (2016). Nutrition in Pregnancy: Optimising Maternal Diet and Fetal Adaptations to Altered Nutrient Supply. Nutrients.

[24] Marques A.H., O’Connor T.G., Roth C., Susser E., Bjørke-Monsen A.L. (2013). The influence of maternal prenatal and early childhood nutrition and maternal prenatal stress on offspring immune system development and neurodevelopmental disorders. Front. Neurosci..

[25] Gernand A.D., Schulze K.J., Stewart C.P., West K.P., Christian P. (2016). Micronutrient deficiencies in pregnancy worldwide: Health effects and prevention. Nat. Rev. Endocrinol..

[26] Borge T.C., Aase H., Brantsæter A.L., Biele G. (2017). The importance of maternal diet quality during pregnancy on cognitive and behavioural outcomes in children: A systematic review and meta-analysis. BMJ Open.

[27] Fadare O., Amare M., Mavrotas G., Akerele D., Ogunniyi (2019). Mother’s nutrition-related knowledge and child nutrition outcomes: Empirical evidence from Nigeria. PLoS ONE.

[28] Lindqvist M., Lindkvist M., Eurenius E., Persson M., Ivarsson A., Mogren I. (2016). Leisure time physical activity among pregnant women and its associations with maternal characteristics and pregnancy outcomes. Sex Reprod. Healthc..

[29] Özlem A., Rathfisch G. (2016). Effect of lifestyle interventions of pregnant women on their dietary habits, lifestyle behaviors, and weight gain: A randomized controlled trial. J. Health Popul. Nutr..

[30] Desta M., Akibu M., Tadese M., Tesfaye M. (2019). Dietary Diversity and Associated Factors among Pregnant Women Attending Antenatal Clinic in Shashemane, Oromia, Central Ethiopia: A Cross-Sectional Study. J. Nutr. Metab..

[31] Aynaci G. (2019). Nutrition perspective from the view of pregnant women: Their understanding of fetal well-being relative to their diet. Prog. Nutr..

[32] Most J., Rebello C.J., Altazan A.D., Martin C.K., St Amant M., Redman L.M. (2019). Behavioral Determinants of Objectively Assessed Diet Quality in Obese Pregnancy. Nutrients.

[33] European Food Safety Authority (2017). Dietary Reference Values for Nutrients Summary Report.

[34] Jarosz M. (2017). Nutrition Standards for the Polish Population.

[35] Rasmussen K.M., Yaktine A.L. (2009). Weight Gain During Pregnancy: Re-Examining the Guidelines.

[36] Hanson M.A., Bardsley A., De-Regil L.M., Moore S.E., Oken E., Poston L., Ma R.C., McAuliffe F.M., Maleta K., Purandare C.N. (2015). The International Federation of Gynecology and Obstetrics (FIGO) recommendations on adolescent, preconception, and maternal nutrition: “Think Nutrition First”. Int. J. Gynaecol. Obstet..

[37] Kaiser L.L., Campbell C.C., Academy Positions Committee Workgroup (2014). Practice paper of the Academy of Nutrition and Dietetics abstract: Nutrition and lifestyle for a healthy pregnancy outcome. J. Acad. Nutr. Diet..

[38] Pieszko M., Ciesielska-Piotrowicz J., Skotnicka M., Małgorzewicz S. (2017). Health behaviors of pregnant women with higher and basic education—Preliminary research. Pediatr. Med. Rodz..

[39] Suliga E. (2015). Nutritional behaviours of pregnant women in rural and urban environments. Ann. Agric. Environ. Med..

[40] Kobiołka A., Goraus M., Mężyk I. (2015). Effect of pregnancy in to change eating habits women of childbearing age. Zdrow. Dobrost..

[41] EFSA (2012). Scientific Opinion on the Tolerable Upper Intake Level of eicosapentaenoic acid (EPA), docosahexaenoic acid (DHA) and docosapentaenoic acid (DPA). EFSA J..

[42] Hyżyk A.K., Sokalska N. (2011). Assessment of weight changes in pregnant women. Now. Lek..

[43] Kobus-Cisowska J., Kmiecik D., Przeor M., Jędrusek-Golińska A., Waszkowiak K., Żołna H. (2016). Assessment of the level of nutritional knowledge and diet of women during pregnancy. Bromat. Chem. Toks..

[44] Joint FAO/WHO Export Consultation on Diet NatPoCD 92003 (2003). Diet, Nutrition and the Prevention of Chronic Diseases.

[45] Przybyłowicz K.E., Janiszewska K., Przybyłowicz M., Grzybiak M. (2012). Relationship between the pre-pregnancy BMI, intake of fibre and fat during pregnancy and the birth weight of neonates. Bromat. Chem. Toks..

[46] Food and Agriculture Organization of the United Nations, World Health Organization (2001). Vitamin and Mineral Requirements in Human Nutrition.

[47] Abd-Elmohdy Emara H. (2019). Effect of Nutrition Education Package on Pregnant Women Knowledge and Healthy Dietary Practice. IOSR JNHS.

[48] European Market Observatory for Fisheries and Aquaculture Products (EUMOFA) (2016). The EU Fish Market.

[49] Hryszko K. (2018). Rynek Ryb. Stan i Perspektywy. Analizy Rynkowe.

[50] Godala M., Pietrzak K., Łaszek M., Gawron-Skarnek A., Szatko F. (2012). Health behaviours of pregnant residents of Łódź. Part I. Diet and vitamin-mineral supplementation. Probl. Hig. Epid..

[51] Trumbo P., Schlicker S., Yates A.A., Poos M. (2002). Dietary reference intakes for energy, carbohydrate, fiber, fat, fatty acids, cholesterol, protein and amino acids. J. Am. Diet. Assoc..

[52] EFSA (2012). EFSA Panel on Dietetic Products, Nutrition and Allergies (NDA). Scientific Opinion on Dietary Reference Values for protein. EFSA J..

[53] World Health Organization (2015). Guideline: Sugars Intake for Adults and Children.

[54] World Health Organization Benefits of Regular Physical Activity. http://www.euro.who.int/en/health-topics/disease-prevention/nutrition/a-healthy-lifestyle/benefits-of-regular-physical-activity.

[55] World Health Organization Global Recommendations on Physical Activity for Health: 18–64 Years Old. http://www.who.int/dietphysicalactivity/physical-activity-recommendations-18-64years.pdf?ua=1.

[56] National Institute for Health and Clinical Excellence (2010). Dietary Interventions and Physical Activity Interventions for Weight Management before, during and after Pregnancy.

[57] Padmapriya N., Shen L., Soh S.E., Shen Z., Kwek K., Godfrey K.M., Gluckman P.D., Chong Y.S., Saw S.M., Müller-Riemenschneider F. (2015). Physical activity and sedentary behavior patterns before and during pregnancy in a multi-ethnic sample of Asian women in Singapore. Matern. Child Health J..

[58] Fazzi C., Saunders D.H., Linton K., Norman J.E., Reynolds R.M. (2017). Sedentary behaviours during pregnancy: A systematic review. Int. J. Behav. Nutr. Phys. Act..

[59] Lardon E., St-Laurent A., Babineau V., Descarreaux M., Ruchat S.M. (2018). Lumbopelvic pain, anxiety, physical activity and mode of conception: A prospective cohort study of pregnant women. BMJ Open.

